# Effects of Balance Exercise Interventions on Balance-Related Performance in People With Multiple Sclerosis: A Systematic Review and a Meta-Analysis of Randomized Controlled Trials

**DOI:** 10.1177/15459683241273402

**Published:** 2024-08-20

**Authors:** Andreas Wallin, Sverker Johansson, John Brincks, Ulrik Dalgas, Erika Franzén, Jacob Callesen

**Affiliations:** 1 Department of Neurobiology, Care Sciences and Society, Division of Physiotherapy, Karolinska Institutet, Stockholm, Sweden; 2 Women’s Health and Allied Health Professionals Theme, Medical Unit Allied Health Professionals, Karolinska University Hospital, Stockholm, Sweden; 3 Faculty of Health Science, Research Centre for Prevention and Health Promotion, VIA University College, Aarhus, Denmark; 4 Department of Public Health—Exercise Biology, Aarhus University, Aarhus, Denmark

**Keywords:** balance training, exercise, intervention, multiple sclerosis, rehabilitation, systematic review

## Abstract

**Background:**

Balance training covers a range of different modalities and complexity levels for people with multiple sclerosis (MS). When evaluating the effects of balance training across different kinds of interventions, determination of the specific intervention content that predict effects are needed.

**Objective:**

To investigate the effects of balance training on gait and dynamic balance outcomes.

**Methods:**

Four databases were systematically searched. Randomized controlled trials involving people with MS (Expanded Disability Status Scale [EDSS] score ≤7.5) where at least 50% of the intervention targeted balance control were included. Interventions were categorized based on training types. Risk-of-bias was assessed using the Tool for the Assessment of Study Quality and Reporting in Exercise (TESTEX).

**Results:**

A total of 18 included studies involved 902 people with MS (EDSS range from 0 to 7.5). Interventions evaluated with a balance composite score or a mobility test showed a moderate effect size (ES = 0.46 [95% confidence interval (CI) = 0.18 to 0.74]; *p* < .01) and a small overall ES (ES = 0.19 [95% CI = 0.01–0.36]; *p* = .04), respectively, across different training types. Stepping and gait speed outcomes showed no effect. Cognitive dual-task training showed a significant effect (ES = 0.81 [95% CI = 0.24 to 1.37]) on subgroup level, when evaluated with a mobility outcome measure. The median TESTEX score on study quality and reporting was 11 (maximum score = 15).

**Conclusions:**

Improvements of balance were found across interventions when measured by balance composite scores and mobility tests, but not when measured by stepping or gait speed outcomes. Large training volume was positively associated with effect on balance. A definition of intensity in balance training is needed for evaluation of its impact on the effect of balance interventions.

## Introduction

Multiple sclerosis (MS) is a chronic inflammatory and neurodegenerative disease of the central nervous system.^1^ As the disease progresses, a wide range of impairments appear, including disturbances in gait and balance.^1,2^ About 50% to 80% of people with MS (PwMS) live with balance impairment,^2^ resulting in decreased mobility, and increased fall risk.^3-5^

Balance control is a complex motor skill derived from interaction of multiple subsystems^6^ which includes motor, sensory, and cognitive balance components (Table 1). Neural lesions and degeneration due to MS affect signal conduction and integration involving peripheral nerves,^7,8^ spinal cord, and cerebral cortex.^9^ Degeneration of neural pathways alters the integration of sensory inputs and hampers the execution of rapid, adequate motor output^10^ causing deficits of balance control.^11^ Furthermore, the cognitive processing of sensory inputs is also affected. This processing is influenced by, for example, limbic activity,^12^ fatigue,^13^ anticipation,^14^ and divided attention.^15-17^

**Table 1.** Definition of Concepts.

Concept	Definition
Balance	The act of maintaining, achieving, or restoring a state of control during any posture^11^
Balance control	The ability to control the center of mass relative to the base of support (steady-state, proactive, and reactive)^18^
Balance training	Exercises that are designed to improve an individual’s ability to withstand challenges from postural sway or destabilising stimuli caused by self-motion, the environment, or other objects^19^
Cognitive components	Balance components challenging divided attention with additional motor or cognitive tasks (ie, dual-tasking)^20^
Dynamic balance	The ability to maintain balance while performing movements or actions that require displacing or moving oneself (no reference)
Exercise	Exercise is physical activity that is planned, structured, repetitive, and purposive in the sense that improvement or maintenance of 1 or more components of physical fitness is an objective^21^
Gait (as outcome)	Manner of walking or moving on foot measured either quantitatively or qualitatively (no reference)
Motor components	Balance components challenging limits of stability, anticipatory motor strategies, reactive motor strategies, and control of dynamics^20^
Motor performance	Skill when performing tasks that require precise control of movement^18^
Motor task	Any task that involves the movement of a body^18^
Sensory components	Balance components challenging sensory systems such as the vestibular, visual, and somatosensory systems (sensory strategies)^20^
Training type	Cognitive Dual-task, Sensory-Motor Integration, Task-Oriented Balance, and Exergame^20^

Balance training is defined as exercises focusing on controlling the center of mass relative to the base of support during different challenging activities. The causal effect from balance training is explained by neuroplasticity, defined as “*the ability of the central nervous system to adapt in response to changes in the environment or lesions*.”^22^ The strive for an improved quality of performance when conducting a challenging motor task promotes reorganization in neural networks,^18^ leading to improvement of balance. To enhance balance performance balance training should challenge the subsystems^6^ underlying balance control and aim to enhance balance for at least 50% of the intervention,^19^ where most exercises should focus on improving sensorimotor processes of balance control.

When outlining protocols for resistance and aerobic training, it is recommended to adhere to the frequency, intensity, type, time, volume, and progression principles, which encompasses Frequency (how often), Intensity (how hard), Time (duration), and Type (mode of exercise), Volume (amount of exercise), and Progression (how exercise is advanced).^23^ Training volume can be calculated by multiplying *Frequency/number of sessions* by *Time per training session* and/or *Duration of intervention*. Of the mentioned parameters, intensity is significantly associated with improvement in resistance training and aerobic training. However, in balance training protocols, *intensity* is often conflated with training volume. This contrasts with its usage in resistance and aerobic training, where intensity is typically expressed as repetition maximum or as percent of maximum heart rate (%HR_max_) or heart rate reserve (%HR_max_-resting), respectively.^23^ In these contexts, intensity represents a relative load, with the individual’s maximal capacity defining the absolute scale range. However, in balance training, there’s no established expression of intensity that accounts for the challenge to the balance control relative to the individual’s capacity. Yet, Farlie et al^24^ has proposed a definition where “*the degree of challenge to the balance control system relative to the capacity of the individual to maintain balance*” can be considered as intensity.

Balance training is carried out in approaches ranging from static position training and functional training during ambulation to vestibular-ocular training, horse riding, Tai Chi, and exergaming. Therefore, the substantial diversity between these interventions poses a specific challenge when recommending 1 type over another.

A recent review indicates the importance of training volume in achieving effective results in diverse interventions that address balance in PwMS.^25^ Balance training research performed in healthy elderly populations suggests that *duration* of training and *level of challenge* (intensity), optimally progressive, are critical factors for inducing beneficial training effects. This recommendation is asserted universally, disregarding divergent contributions from different types of balance training^26^ and without a common understanding of what intensity in balance training is. Nevertheless, the impact of content in different balance training types to improve balance remains unclear.

A former review by Brincks et al^20^ categorized interventions according to their specific targeting of motor, sensory, and cognitive components (Table 1). The number of components included in the training was suggested to reflect the level of intervention complexity. The review proposed an approach where complexity mirrors intensity, and revealed that several studies across training types challenged participants at a moderate level. The review also concluded that the effect on balance control was evaluated with many outcome measures, ranging from measurements of single properties (eg, sway area in standing) to functional composite scores such as the Bergs balance Scale. However, the review neither investigated whether an effect of balance training on balance control was evident nor if the level of complexity of an intervention impacted the effects of the balance intervention. Thus, the present systematic review aimed to investigate the effects of diverse balance training interventions on gait and dynamic balance outcomes and if intervention complexity impacts the effect of a balance training intervention in PwMS.

## Methods

### Protocol and Registration

This systematic review was preregistered in the international prospective register of systematic reviews (PROSPERO:CRD42020181093), the protocol is available at https://www.crd.york.ac.uk/prospero/display_record.php?RecordID=181093. The review follows the guidelines presented in the Preferred Reporting Items for Systematic Reviews and Meta-analysis (PRISMA) statement.^27^

### Eligibility Criteria

Eligible study designs were randomized controlled trials (RCT) involving adults aged 18 years or older with a diagnosis of MS, an Expanded Disability Status Scale (EDSS) ≤7.5,^28^ able to transfer from 1 seated position to another independently (eg, from a wheelchair to a chair), not considering disease duration and phenotype of MS. Planned and structured balance training interventions aimed at improving sensorimotor processes of balance control for at least 50% of the total intervention time, and which challenged at least 2 out of 6 balance subsystems (ie, biomechanical constraints, movement and sensory strategies, orientation in space, control of dynamics, and cognitive processing^6^) were included.

Studies had to include non-training controls (usual care) or active control conditions that included exercise interventions that had no expected effects on balance control, for example, stretching or relaxation. Studies were eligible for inclusion if balance control was evaluated with outcomes reflecting postural stability (such as Berg Balance Scale, Functional Reach Test, Tandem Stand Test, Romberg’s Test, and measures of postural sway) or activities challenging balance during walking or functional mobility (such as Mini-Balance Evaluation System Test [BESTest], Six Spot Step Test, Functional Gait Assessment, Timed Up & Go, 25-Foot Walk Test, 10-Meter Walk Test, and 6-Minute Walk Test).

### Searches, Information Sources, and Study Selection

**Figure 1.**
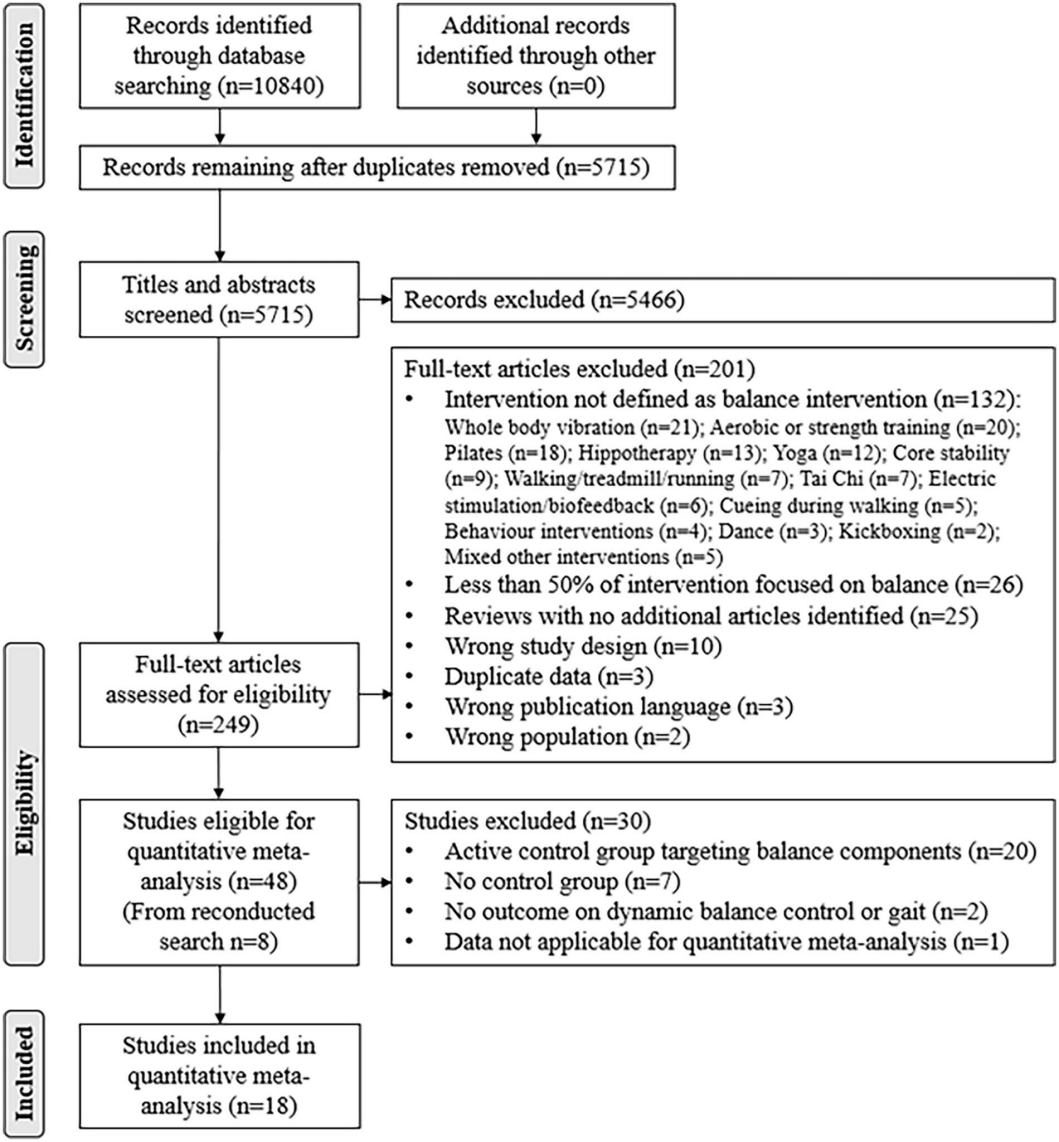
Preferred reporting items for systematic reviews and meta-analyses flow diagram on the search result and study selection process.

This study is a continuation of our previous systematic review.^20^ The review included 40 studies based on a literature search conducted in April 2020 (Figure 1). For the present review the literature search was updated in January 2023, and new studies were added to the sample of studies from the previous review.^20^ The searches were conducted by librarians at the Karolinska Institutet University Library in the following databases: Medline (Ovid), EMBASE, Web of Science (Clarivate), and Cinahl (Ebsco). Search terms including Medical Subheadings, Emtree terms, and Cinahl headings were MS, postural balance, walking, gait, clinical trials, controlled trials, randomized controlled trials, and case-controlled trials. Full search strategy is available at https://www.crd.york.ac.uk/PROSPEROFILES/181093_STRATEGY_20200420.pdf. Screening process on newfound studies was performed by the same authors (J.B. and S.J.) as in the previous review.^20^ Eligibility assessment for final inclusion of studies in the present review was conducted by 2 other authors (A.W. and J.C.).

### Data Management and Collection Process

Data extraction included study design and population, sample size, training and control intervention type, duration, volume, and outcome measures (Table 2). Interventions were grouped into 4 *training types* (Cognitive Dual-task, Sensory-Motor Integration, Task-Oriented Balance, and Exergame) based on the main focus and balance components (motor, sensory, and cognitive) challenged during the intervention. Categorization of training types and challenged balance components in the interventions was adapted from the previous review^20^ presented in Table 2. Balance-related outcome measures were grouped into 4 Outcome categories based on type of outcome measure or the specific domain evaluated. The outcome categories were: Balance composite score (Berg Balance Scale and mini-BESTest); Mobility (Timed Up&Go and Six Spot Step Test); Gait speed outcomes (10-Meter Walk Test, 25-Foot Walk Test, and gait speed as a parameter measured in gait analysis); and Stepping (Four Square Step Test; Table 2). Two reviewers (A.W. and J.C.) completed the data extraction. Missing data were requested from authors of 7 studies^29-35^ and obtained from 5 of them.^30,31,33-35^ Data were considered missing if the author had not replied after 2 follow-up requests. Missing data on mean values and standard deviation from 1 study were calculated based on reported values on 95% confidence interval in the study.^32^

**Table 2.** Descriptive Data of the Included Balance Training Interventions in People With Multiple Sclerosis: Information on Training Category, Study Design, Study Population, Disease Characteristics, and Functional Mobility Level.

Author	Training category	Intervention duration (weeks)	Design	n/Female:Male	Age, years (SD or min;max)	Years since diagnosis (SD or min;max)	EDSS	Disease course (RR/SP/PP)	Functional mobility level
Callesen et al. 2020	Sensory-motor integrated	10	Multi-center RCT	I: 28/23:5 C: 20/16:4	I: 51 (31;75) C: 56 (30;73)	I:10 (2;33) C: 11 (1;32)	2-6.5	I: 21/4/3 C: 13/3/4	Six Spot Step Test score >8 s or 25-Foot Walk Test >5 s
Carling et al. 2017	Sensory-motor integrated	7	Multi-center RCT	I: 25/19:6 C: 26/16:10	I: 61.6 (11.3) C: 54.7 (8.2)	I: 21.6 (13.8) C: 20.2 (10.4)	4-7.5	I: 0/17/8 C: 6/15/5	Ability to transfer between sitting positions
Cattaneo et al. 2018	Sensory-motor integrated	7	Multi-center RCT	I: 78/54:24 C: 41/29:12	I: 48.9 (11.1) C: 46.7 (11.4)	I: 14.0 (8.6) C:12.9 (10.4)	NR	NR	Ability to walk with aid ≥6 m and stand (open eyes) for ≥30 s
Eftekharsadat et al. 2015	Exergame	12	RCT	I: 15/10:5 C: 15/12:3	I: 33.4 (8.1) C: 37.0 (8.3)	I: 5.8 (3.9) C: 8.3 (4.3)	NR	Either RR or SP	Ability to stand for ≥20 min on the Biodex Balance System
Felippe et al. 2019	Cognitive dual-task	26	Single-blind RCT	I: 13/3:10 C: 14/5:9	I: 35 (16.5) C: 38 (15.3)	I: 5 (3.0) C: 8 (7.3)	0-5	NR	NR
Forsberg et al. 2016	Sensory-motor integrated	7	RCT	I: 35/28:7 C: 38/31:7	I: 52 (10) C: 56.3 (11)	I: 15 (9) C:16 (11)	NR	I: 20/11/4 C: 13/20/5	Ability to walk 100 m, get up from the floor, unable to stand in tandem stance for 30 s
Hebert et al. 2018	Sensory-motor integrated	6	RCT	I: 44/37:7 C: 44/38:6	I: 46.5 (8.8) C: 43.0 (10.8)	I: 8.3 (5.7) C: 8.5 (7.6)	NR	NR	EDSS score ≤ 6
Hoang et al. 2016	Exergame	12	RCT	I: 28/21:7 C: 22/17:5	I: 53.4 (10.7) C: 51.4 (12.8)	I: 11.6 (9.1) C: 13.4 (6.9)	2-6	I: 15/5/8 C: 11/7/2 (2 unknown)	Ability to stand independently without personal support and to complete the step training
Hortobágyi et al. 2022	Exergame (e) and Task-oriented (t)	5	RCT	I(e): 14/12:2 I(t): 14/12:2 C: 12/11:1	I(e): 48.2 (5.5) I(t): 46.9 (6.5) C: 44.4 (6.8)	I(e): 12.1 (2.7) I(t): 13.6 (4.1) C: 14.0(4.1)	5-6	I(e): 7.7/0/14 I(t): 5.9/0/14 C: 4.8/0/12	EDSS score 4-6
Nilsagaard et al. 2013	Exergame	6	Multi-center RCT	I: 42/32:10 C: 42/32:10	I: 50 (11.5) C: 49.4 (11.1)	I: 12.5 (8) C: 12.2 (9.2)	NR	I: 26/13/3 C: 28/13/1	Ability to walk 100 m without resting
Novotna et al. 2019	Exergame	4	RCT	I: 23/17:6 C: 16/12:4	I: 39.4 (9.7) C: 42.6 (10.6)	I: 15 (8.6) C: 14.5 (9.9)	1.5-7	NR	Ability to walk with or without walking aid ≥5 m and to stand ≥10 min
Ozkul et al. 2020	Task-oriented	6	RCT	I: 10/6:4 C: 10/6:4	I: 46.0 (29-47)^a^ C: 41.5 (28-47)^a^	I: 16 (7-21)^a^ C:13.5 (7-20)^a^	2-5.5	I: 6/0/4 C: 6/0/4	No explicit criteria for entering the study
Ozkul et al. 2023	Cognitive dual-task (c) and Task-oriented (t)	6	RCT	I(c): 13/10:3 I(t): 13/11:2 C: 13/10:3	I(c): 34.3 (12.1) I(t): 36.3 (11.6) C: 35.6 (10.4)	I(c): 4.2 (2.2) I(t): 4.8 (2.3) C: 5.1 (4.1)	0-2	I(c): 13/0/0 I(t): 13/0/0 C: 13/0/0	EDSS score < 2, and self-reported concerns about dual-task
Prosperini et al. 2013	Exergame	12	RCT (cross-over)	I: 18/13:5 C: 18/12:6	I: 35.3 (8.6) C: 37.1 (8.8)	I: 12.2 (6.0) C: 9.3 (5.3)	1.5-5	Either RR or SP	Ability to walk without rest for ≥100 m; the presence of objective balance disturbance
Robinson et al. 2015	Exergame (e) and Sensory-motor integrated (s)	4	RCT	I(e): 20/14:6 I(s): 19/12:7 C: 17/12:5	I(e): 52.6 (6.1) I(s): 53.9 (6.5) C: 51.9 (4.7)	NR	≤6	NR	Self-reported ability to walk 100 meters with or without resting with walking aid
Straudi et al. 2014	Task-oriented	2	Pilot RCT	I: 12/7:5 C: 12/10:2	I: 49.9 (7.5) C: 55.2 (13.8)	I: 12.2 (6.9) C:18.2 (9.5)	4-5.5	I: 4/3/5 C: 2/5/5	Ability to walk ≥100 m with no constant assistive device required
Straudi et al. 2022	Task-oriented	2	RCT	I: 18/11:7 C: 18/12:6	I: 49.7 (13.6) C: 52.6 (12.6)	I: 12.4 (11.4) C: 9.64 (5.6)	4-5.5	I: 8/5/5 C: 7/5/6	EDSS score 4-5.5. Ability to walk 100-500 m without aid
Yazgan et al. 2020	Exergame Wii Fit (w) and Exergame Balance Trainer (b)	8	RCT	I(w): 15/13:2 I(b): 12/12:0 C: 15/13:2	I(w): 47.5 (10.5) I(b): 43.1 (8.7) C: 40.7 (8.8)	I(w): 12.1 (6.6) I(b): 14.9 (6.5) C: 11.1 (5.7)	2.5-6	I(w): 11/1/3 I(b): 8/1/3 C: 14/0/1	Ambulatory participants were included
Weighted mean across studies (SD)					47.5 (6.6)	12.2 (3.7)			

Abbreviations: n, sample size; SD, standard deviation; min, minimum; max, maximum; EDSS, Expanded Disability Status Scale; RR, relapsing-remitting Multiple Sclerosis; SP, secondary progressive Multiple Sclerosis; PP, primary progressive Multiple Sclerosis; RCT, randomized controlled trial; I, intervention group; C, control group; sec, seconds; NR, not reported; m, meter; min, minutes; (e), Exergame training; (t), Task-oriented training; (c), Cognitive dual-task training; (s), Sensory-motor integrated training; (w), Exergame Wii Fit; (b), Exergame Balance Trainer.

a Inter-Quartile Range.

### Risk-of-Bias Assessment

Risk-of-bias was independently assessed by 2 blinded reviewers (A.W. and J.C.) using the Tool for the assEssment of Study qualiTy and reporting in EXercise (TESTEX) scale.^36^ The TESTEX scale is considered a reliable tool specifically designed to facilitate the reviewing of exercise training trials.^36^ The TESTEX scale consists of 12 items, of which 5 refer to the assessment of study quality (items 1 to 5), and 7 refer to the assessment of study reporting (items 6 to 12). The TESTEX scale ranges from 0 to 15. After blinded assessment (A.W. and J.C.), disagreements were discussed and resolved with the other review authors (S.J., J.B., U.D., and E.F.). In TESTEX scale item 2 (randomization), minimization was considered a valid randomization method, while cluster randomization was not. Furthermore, in TESTEX scale item 7 (intention-to-treat analysis), imputation of missing data was accepted as a valid intention-to-treat approach.

### Synthesis of Results

Data from the included studies were descriptively summarized (Table 2). Random-effects meta-analyses based on data from functional balance outcome measures were conducted using IBM^®^ SPSS^®^ Statistics version 28.

Outcome measures capturing comparable aspects of functional balance control were pooled into specific outcome categories. Thus, interventions evaluated using multiple outcome measures could be included in meta-analysis within multiple outcome categories.

Post-intervention mean and standard deviation values of different outcome measures were used to calculate the intervention effect, as recommended by the Cochrane Handbook of Meta-analysis.^37^ Values from midpoint measurements were used in studies with cross-over^38^ and/or wait-list^39^ RCT designs. Extracted baseline values were also displayed, although not used in the analysis. This was done to ensure full transparency regarding discrepancies between the original studies’ results and those reported in this review. The pooled effect was summarized using Hedges’ *g* statistics to allow for comparison across different outcome measures. Forest plots illustrated effect sizes (ES) with 95% confidence intervals (CIs) for individual studies and pooled effect.

Positive ES indicated improvement in favor of the intervention. An ES > 0.61 was regarded as large, >0.31 and ≤0.61 as moderate, and >0.14 and ≤0.31 as a small.^40^ *p*-values <.05 were considered significant.

Heterogeneity was defined as Higgins’ *I*^2^ > 50%, and no or limited heterogeneity was defined as Higgins’ *I*^2^ ≤ 50%.^41^

Funnel plots were created to assess publication bias in outcome categories, including at least 10 studies.^42^

Univariate meta-regression analyses were conducted post hoc to investigate whether training volume and/or training complexity (number of balance components included in the training) were associated with ES. Training volume was log-transformed to account for a skewed distribution.

Additional adjusted meta-analyses were conducted where baseline values were retracted from the group-level post-measures. Standard deviations from post-intervention measures were used. This was done to adjust for potential misinterpretations that changes between post-intervention measures (intervention versus control) were caused by changes between groups when, in fact, they were due to a difference between baseline measures. Additional analyses are presented in the Supplemental Material.

## Results

### Study Selection and Characteristics

The updated search yielded 2932 new records. After removal of duplicates, 690 records were screened for inclusion based on title and abstract (Figure 1). From this, 8 full-text articles were added to the sample from the previous review,^20^ resulting in a total of 48 studies eligible for the present review, of which, 18 studies were included for meta-analysis (Figure 1).

The studies included 902 PwMS with an EDSS score of 0 to 7.5 (Table 2). In the studies where the EDSS level was not reported,^30,35,43-45^ it was noted that functional mobility levels of included PwMS matched the EDSS range of the other studies. The weighted mean age of PwMS samples across studies was 47.5 (SD = 6.6) years, ranging from 33.4^32^ to 61.6.^31^ The weighted mean disease duration (years since diagnosis) of PwMS samples across studies was 12.2 (SD = 3.7) years, ranging from 4.2^46^ to 21.6^31^ years (Table 2).

### Training Types and Outcome Categories

Six studies applied the training type sensory-motor integrated training,^30,31,43,45,47,48^ 8 applied exergame training,^34,35,38,44,48-51^ 2 applied cognitive dual-task training,^32,46^ and 5 studies applied task-oriented training.^33,34,39,46,52^ Three studies included interventions that comprised 2 different training types^34,46,48^ (Table 2).

The duration of the interventions ranged from 2^39,52^ to 26 weeks^32^ (Table 2), with a total delivered training volume ranging from 6^35^ to 52^32^ hours (Table 3). In cognitive dual-task training and sensory-motor integrated training, the median number of balance components challenged was 6.5, while the median number of components was 6 within the task-oriented training. The median number of components challenged in exergame training was 3 (Table 3).

**Table 3.** Overview of Intervention Types, Outcome Categories, Balance Components Challenged in the Training, Training Volume in the Intervention, and the Corresponding Effect Size.

Training type	Author	Outcome category	Balance components challenged in the training (median)										Training volume (hours)	Effect size
Cognitive dual-task	Ozkul et al. 2023^a^	Mobility	M	M	M	S	S	S	C	C		(6.5)	12	0.89**
	Felippe et al. 2019	Mobility	M	M	M	S	C						52	0.73
Sensory-motor integrated	Callesen et al. 2020	Composite	M	M	M	M	S	S	S	C	C	(6.5)	20	0.50
		Gait Speed											20	0.41
		Mobility											20	0.36
	Forsberg et al. 2016	Composite	M	M	M	S	S	S	C	C			14	0.75**
		Mobility											14	0.38
	Cattaneo et al. 2018	Composite	M	M	M	S	S	S	C	C			12	0.06
	Carling et al. 2017	Composite	M	M	M	S	S	S	C				14	0.08
		Mobility											14	−0.20
	Hebert et al. 2018	Gait Speed	M	M	M	S	S	S	C				12	−0.27
	Robinson et al. 2015^a^	Gait Speed	M	M	S								7	0.11
Task-oriented	Hortobágyi et al. 2022^a^	Composite	M	M	M	M	S	S	C	C		(6)	25	1.20**
	Ozkul et al. 2023^a^	Mobility	M	M	M	S	S	S					12	0.33
	Ozkul et al. 2020	Composite	M	M	M	S	S	S					12	0.30
		Mobility											12	0.21
	Straudi et al. 2014	Mobility	M	M	M	S							20	0.70
		Gait Speed											20	0.45
	Straudi et al. 2022	Mobility	M	M	M	S							20	0.06
		Gait Speed											20	−0.14
Exergame	Hortobágyi et al. 2022^a^	Composite	M	M	M	M	S	S	C			(3)	25	1.39**
	Hoang et al. 2016	Mobility	M	M	M	M	S	C					12	−0.13
		Gait Speed											12	0.13
	Novotna et al. 2019	Composite	M	M	S	C							7	−0.08
		Mobility											7	−0.28
	Prosperini et al. 2013	Gait Speed	M	M	S								30	0.30
		Stepping											30	0.28
	Yazgan et al. 2020	Composite	M	M	S								16	0.62
		Mobility											16	0.00
	Eftekharsadat et al. 2015	Composite	M	M	S								8	0.44
		Mobility											8	0.44
	Robinson et al. 2015^a^	Gait Speed	M	M	S								7	0.00
	Nilsagaard et al. 2013	Gait Speed	M	M	S								6	0.08
		Mobility											6	0.00
		Stepping											6	−0.09

Outcome category: Mobility = Timed Up&Go, Six Spot Step Test; Gait Speed = 10-Meter Walk Test, 25-Foot Walk Test; Stepping = Four Square Step Test; Composite = Berg Balance Scale, Mini-BESTest.

Balance components challenged in the training: M = Motor components (limits of stability, anticipatory motor strategies, reactive motor strategies, and control of dynamics); S = Sensory components (vestibular, visual, and somatosensory systems); C = Cognitive components (motor or cognitive dual-task).

a The study includes 2 training types.

** Significant *p* < .05.

Two studies reported 3 outcome measures of functional balance,^35,47^ which enabled inclusion into 3 different outcome categories in the meta-analyses (Table 3). Further, 10 studies were included into 2 outcome categories,^30,31,33,38,39,44,49-52^ and 6 studies were evaluated with 1 outcome measure of functional balance^32,34,43,45,46,48^ (Table 3).

### Risk-of-Bias Assessment

The median TESTEX score of included studies was 11 (Table 4). Two studies (11%) reported activity monitoring in control groups^35,46^; 7 studies (39%) performed a valid intention-to-treat analysis^31,39,43,44,48,50,52^; and 9 studies (50%) reported that exercise load was adjusted to keep the relative intensity constant throughout the intervention period.^31,32,34,38,46-48,50,51^ The remaining TESTEX items were reported in more than 50% of the included studies. All studies presented a training protocol enabling calculation of the intended exercise volume. Detailed information on individual study scores can be found in Table 4.

**Table 4.** Item and Total Scores on Risk of Bias Assessment for Each Study Included in the Meta-analysis Based on the Tool for the assEssment of Study qualiTy and Reporting in EXercise (TESTEX) Scale: 1 Point Indicate That Criterion Has Been Met, Zero (0) Point Indicate That Criterion Has Not Been Met.

Section	Study quality item scores					Study reporting item scores										Total score (max 15)
Item	*1*	*2*	*3*	*4*	*5*	*6a*	*6b*	*6c*	*7*	*8a*	*8b*	*9*	*10*	*11*	*12*	
Study	*Eligibility criteria*	*Randomization*	*Allocation concealed*	*Baseline data*	*Blinded assessor Primary outcome*	*Outcome measures* in >85% of patients	*Adverse events reported*	*Exercise attendance reported*	*Intention-to-treat analysis*	*Between-group Stats Primary outcome*	*Between-group Stats Secondary outcomes*	*Outcomes point estimates*	*Control Physical activity*	*Exercise load titrated*	*Exercise volume can be calculated*	
Callesen et al. 2020	1	0	1	1	1	1	1	1	0	1	1	1	0	1	1	12
Carling et al. 2017	1	1	1	1	1	1	1	1	1	1	1	1	0	1	1	14
Cattaneo et al. 2018	1	1	1	1	1	1	0	0	1	1	1	1	0	0	1	11
Eftekharsadat et al. 2015	0	1	1	1	1	1	0	0	1	1	1	1	0	0	1	10
Felippe et al. 2019	1	0	1	1	0	1	1	0	0	1	1	1	0	1	1	10
Forsberg et al. 2016	1	1	1	0	1	0	1	1	0	1	1	1	0	0	1	10
Hebert et al. 2018	1	1	1	1	1	1	1	1	0	1	1	1	0	0	1	12
Hoang et al. 2016	1	1	1	1	1	1	1	0	0	1	1	1	0	0	1	11
Hortobágyi et al. 2022	1	1	1	1	1	1	0	0	0	0	0	0	0	1	1	8
Nilsagaard et al. 2013	1	1	1	1	1	1	1	1	0	1	1	1	1	0	1	13
Novotna et al. 2019	1	0	1	1	0	1	1	1	1	0	0	1	0	1	1	10
Ozkul et al. 2020	1	0	1	1	1	1	1	1	0	0	0	1	0	0	1	9
Ozkul et al. 2023	1	0	1	1	1	1	1	0	0	1	1	1	1	1	1	12
Prosperini et al. 2013	1	1	1	1	0	1	1	0	0	1	1	1	0	1	1	11
Robinson et al. 2015	1	1	1	0	0	1	0	0	1	1	1	1	0	1	1	10
Straudi et al. 2014	1	1	1	1	0	1	1	1	1	1	1	1	0	0	1	12
Straudi et al. 2022	1	1	1	1	1	1	0	1	1	0	0	1	0	0	1	10
Yazgan et al. 2020	1	1	0	1	0	1	1	1	0	1	1	1	0	1	1	11
Studies meeting item criterion (%)	94	72	94	89	67	94	72	56	39	78	78	94	11	50	100	

The risk of bias assessment across studies is presented as percentage of studies meeting each item criterion. Items are grouped within respective TESTEX section: *Study quality* and *Study reporting*.

Abbreviation: Stats, statistical comparison.

### Synthesis of Results

#### Effects on Balance Composite Scores

Nine studies that included the training types exergame, sensory-motor integrated, and task-oriented, used a balance composite score for evaluation of the intervention effect^30,31,33,34,43,44,47,50,51^ (Table 5).

**Table 5.** Meta-analysis of the Effect of Balance Training on four Outcome Categories in Subgroups of Training Types.

Outcome category	Training type	Author	Outcome measure	Intervention			Control			Weight (%)	SMD (CI 95%)	Favors	
				n	Mean_pre_ (SD)	Mean_post_ (SD)	n	Mean_pre_ (SD)	Mean_post_ (SD)			Control	Intervention
Balance composite score	Exergame	Yazgan et al. 2020	BBS	15	43.3 (8.3)	49.1 (4.3)	15	44.5 (7.7)	45.4 (7.1)	8.7	.62 (−.12; 1.35)	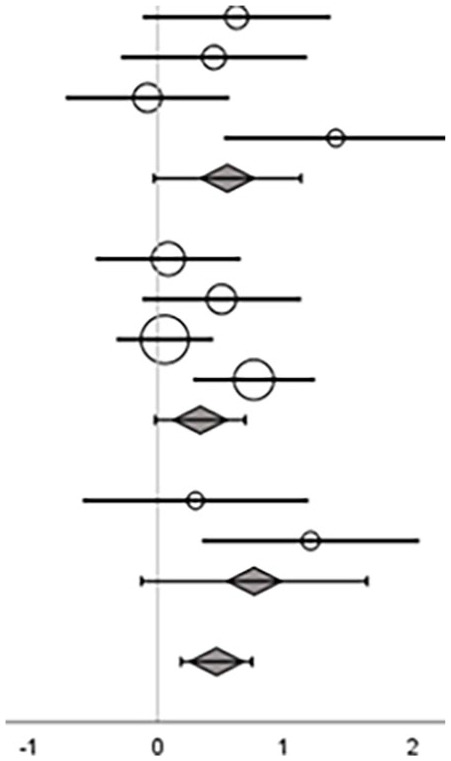	
		Eftekharsadat et al. 2015	BBS	15	51.7 (3.3)	51.9 (3.2)	15	48.5 (9.0)	48.8 (9.0)	8.8	.44 (−.28; 1.17)		
		Novotna et al. 2019	MBT	23	22.4 (6.0)	23.5 (6.0)	16	23.1 (5.2)	23.9 (3.8)	10.2	−.08 (−.72; .56)		
		Hortobágyi et al. 2022	BBS	14	21.7 (3.6)	27.8 (3.8)	12	22.5 (4.4)	22.3 (3.9)	6.9	1.39 (.52; 2.27)		
		Subgroup overall		67			58			34.7	.55 (−.03; 1.12)		
	Sensory-motor integrated	Carling et al. 2017	BBS	23	33.4 (9.7)	37.7 (11.5)	25	36.1 (11.1)	36.7 (11.9)	11.5	.08 (−.48; .65)		
		Callesen et al. 2020	MBT	24	16.6 (6.3)	20.7 (6.4)	18	16.6 (6.0)	17.5 (6.1)	10.5	.50 (−.12; 1.12)		
		Cattaneo et al. 2018	BBS	78	46.6 (9.0)	49.2 (5.3)	41	45.9 (10.8)	48.9 (5.1)	15.8	.06 (−.32; .44)		
		Forsberg et al. 2016	BBS	35	48.9 (5.8)	51.5 (4.5)	38	45.1 (9.0)	46.7 (7.6)	13.5	.75 (.28; 1.23)		
		Subgroup overall		160			122			51.3	.33 (−.02; .69)		
	Task-oriented	Ozkul et al. 2020	BBS	10	37.6 (5.4)	40.8 (5.8)	10	39.1 (5.5)	38.9 (6.4)	6.8	.30 (−.59; 1.18)		
		Hortobágyi et al. 2022	BBS	14	21.9 (2.3)	25.7 (1.1)	12	22.5 (4.4)	22.3 (3.9)	7.2	1.20 (.35; 2.04)		
		Subgroup overall		24			22			14.0	.76 (−.13; 1.64)		
		Total overall		251			202				.46 (.18; .74)		
											Overall effect: *p* = .00		
											Heterogeneity: *I*^2^ = 52%		
Gait speed outcomes	Exergame	Nilsagaard et al. 2013	25FWT	41	6.6 (3.4)	6.2 (3.0)	39	6.5 (3.1)	6.5 (3.8)	19.9	.08 (−.35; .52)	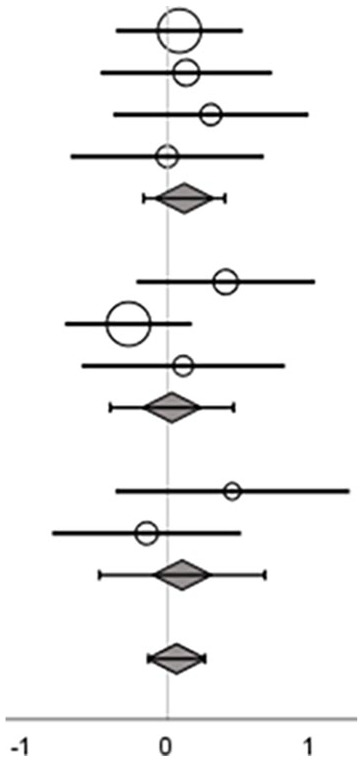	
		Hoang et al. 2016	10MWT	23	12.5 (5.0)	10.5 (4.0)	21	11.4 (4.3)	11.1 (4.9)	10.9	.13 (−.46; .72)		
		Prosperini et al. 2013	25FWT	17	8.5 (2.7)	7.8 (2.8)	17	9.5 (3.3)	8.7 (3.0)	8.4	.30 (−.37; .98)		
		Robinson et al. 2015	GAITRite*	20	.65 (.29)	.77 (.27)	15	.77 (.27)	.77 (.37)	8.5	.00 (−.67; .67)		
		Subgroup overall		101			92			47.7	.12 (−.**16; .40)**		
	Sensory-motor integrated	Callesen et al. 2020	25FWT*	24	1.3 (.3)	1.4 (.3)	18	1.2 (.4)	1.2 (.5)	10.0	.41 (−.21; 1.02)		
		Hebert et al. 2018	25FWT	39	6.2 (2.0)	6.0 (2.0)	42	5.5 (2.0)	5.4 (2.0)	19.9	−.27 (−. 71; .17)		
		Robinson et al. 2015	GAITRite*	16	.76 (.21)	.80 (.24)	15	.77 (.27)	.77 (.37)	7.7	.11 (−.59; .82)		
		Subgroup overall		79			75			37.6	.03 (−.40; .46)		
	Task-oriented	Straudi et al. 2014	10MWT*	12	1.1 (.2)	1.1 (.2)	12	1.0 (.3)	1.0 (.2)	5.8	.45 (−.36; 1.26)		
		Straudi et al. 2022	10MWT*	18	1.3 (.4)	1.5 (.2)	18	1.5 (.3)	1.5 (.2)	8.9	−.14 (−.80; .51)		
		Subgroup overall		30			30			14.7	.10 (−**.47; .68)**		
		Total overall		210			197	.			07 (−.13; .26)		
											Overall effect: *p* = .51		
											Heterogeneity: *I*^2^ = 0%		
Mobility	Cognitive dual-task	Ozkul et al. 2023	TUG	13	6.6 (.7)	5.8 (.6)	13	6.3 (1.0)	6.5 (.8)	4.5	.89 (.08; 1.70)	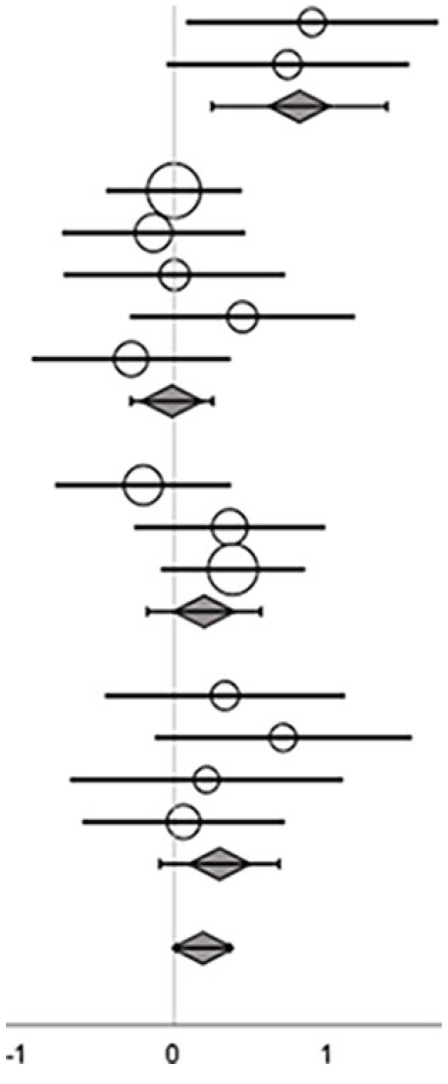	
		Felippe et al. 2019	TUG	13	14.3 (4.9)	13.7 (5.0)	14	17.9 (8.1)	18.6 (7.6)	4.8	.73 (−.05; 1.52)		
		Subgroup overall		26			27			9.2	.81 (.24; 1.37)		
	Exergame	Nilsagaard et al. 2013	TUG	41	12.4 (6.9)	11.4 (5.9)	39	11.3 (5.0)	11.4 (5.7)	14.7	−.00 (−.44; .44)		
		Hoang et al. 2016	TUG	23	13.1 (5.5)	12.3 (4.3)	21	12.1 (3.9)	11.7 (4.6)	8.3	−.13 (−.46; .72)		
		Yazgan et al. 2020	TUG	15	12.3 (5.1)	10.8 (4.9)	15	10.7 (5.9)	10.8 (5.9)	5.7	.00 (− 71; .72)		
		Eftekharsadat et al. 2015	TUG	15	8.7 (2.4)	8.0 (2.2)	15	10.9 (8.3)	11.1 (9.3)	5.6	.44 (−.28; 1.17)		
		Novotna et al. 2019	TUG	23	12.1 (11.5)	11.5 (9.9)	16	9.6 (5.8)	9.2 (5.1)	7.1	−.28 (−.92; .37)		
		Subgroup overall		117			106			41.2	−.01 (−.28; .25)		
	Sensory-motor integrated	Carling et al. 2017	TUG	23	32.5 (25.2)	32.2 (23.6)	25	32.6 (30.5)	27.8 (20.0)	8.0	−.20 (−.77; .37)		
		Callesen et al. 2020	SSST	24	5.3 (1.6)	6.4 (2.0)	18	5.5 (1.7)	5.7 (2.0)	7.6	.36 (−.26; .98)		
		Forsberg et al. 2016	TUG	35	13.7 (5.5)	14.2 (11.4)	38	17.0 (9.1)	18.0 (8.3)	13.2	.38 (−.08; .84)		
		Subgroup overall		82			81			29.8	.19 (−.17; .56)		
	Task-oriented	Ozkul et al. 2023	TUG	13	6.7 (.8)	6.2 (.6)	13	6.3 (1.0)	6.5 (.8)	4.9	.33 (−.45; 1.10)		
		Straudi et al. 2014	TUG	12	10.6 (2.5)	10.1 (1.9)	12	12.1 (4.0)	12.2 (3.8)	4.3	.70 (−.13; 1.53)		
		Ozkul et al. 2020	TUG	10	10.8 (3.4)	9.3 (2.7)	10	9.7 (3.5)	9.9 (2.8)	3.8	.21 (−.67; 1.09)		
		Straudi et al. 2022	TUG	18	10.3 (4.2)	9.0 (2.3)	18	9.7 (2.1)	9.1 (1.4)	6.8	.06 (−.59; .72)		
		Subgroup overall		53			53			19.8	.29 (−.09; .68)		
		Total overall		278			267				.19 (.01; .36)		
											Overall effect: *P* = .04		
											Heterogeneity: *I*^2^ = 6%		
**Stepping**	Exergame	Nilsagaard et al. 2013	FSST	41	16.8 (12.2)	17.2 (16.5)	39	17.7 (13.8)	15.9 (10.7)	70.4	−.09 (−.53; .35)	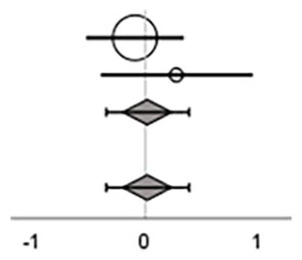	
		Prosperini et al. 2013	FSST	17	17.5 (12.7)	14.8 (10.1)	17	17.4 (9.7)	17.6 (9.5)	29.6	.28 (−.40; .95)		
		Subgroup overall		58			56			100.0	.02 (−**.35; .39)**		
		Total overall		58			56				.02 (−**.35; .39)**		
											Overall effect: *P* = .92		
											Heterogeneity: *I*^2^ = 0%		

SD=Standard Deviation; SMD=Standardized mean difference; CI=Confidence Interval; BBS=Berg Balance Scale; MBT=Mini-BESTest; 25FWT=25-Foot Walk Test measured in seconds; 10MWT=10-Meter Walk Test measured in seconds. *=measured in meters/second; TUG=Timed Up&Go measured in seconds; SSST=Six Spot Step Test measured in 1/seconds; FSST=Four Square Step Test measured in seconds.

Overall ES, across exergaming, sensory-motor integrated training, and task-oriented training showed a moderate effect on balance composite scores, with a considerable heterogeneity (ES = 0.46 [95% CI = 0.18 to 0.74]; *p* = .00, *I*^2^ = 52%; Table 5).

On subgroup level, ES were considered moderate for exergame (ES = 0.55 [95% CI = –0.03 to 1.12]) and sensory-motor integrated training (ES = 0.33 [95% CI = –0.02 to 0.69]), and large for task-oriented training (ES = 0.76 [95% CI = –0.13 to 1.64]). None of the 3 training types reached significance.

#### Effect on Gait Speed Outcomes

Eight studies that included the training types exergame, sensory-motor integrated, and task-oriented applied gait speed as an outcome measure^35,38,39,45,47-49,52^ (Table 5). No overall or subgroup level effect was found in the meta-analysis (ES = 0.07 [95% CI = –0.13 to 0.26]; *p* = .51, *I*^2^ = 0%; Table 5).

#### Effects on Mobility

Thirteen studies that included the training types cognitive dual-task, exergame, sensory-motor integrated, and task-oriented, evaluated the effects of balance training on mobility^30-33,35,39,44,46,47,49,51,52^ (Table 5). Overall, the meta-analysis showed a small significant effect of the interventions on functional balance when evaluated with a mobility test (ES = 0.19 [95%cCI = 0.01 to 0.36]; *p* = .04, *I*^2^ = 6%; Table 5).

At subgroup level, ESs were considered small for sensory-motor integrated (ES = 0.19 [ 95% CI = –0.17 to 0.56]) and task-oriented training (ES = 0.29 [CI 95% = –0.09 to 0.68]), and large for cognitive dual-task training (ES = 0.81 [95% CI = 0.24 to 1.37]), with the latter being significant.

#### Effects on Stepping

Two studies that included the training type exergame applied an outcome category related to stepping for evaluation of the intervention effect^35,38^ (Table 5). No effect was shown (ES = 0.02 [95% CI = –0.35 to 0.39]; *p* = .92, *I*^2^ = 0%).

#### Correlation Analyses

**Figure 2.**
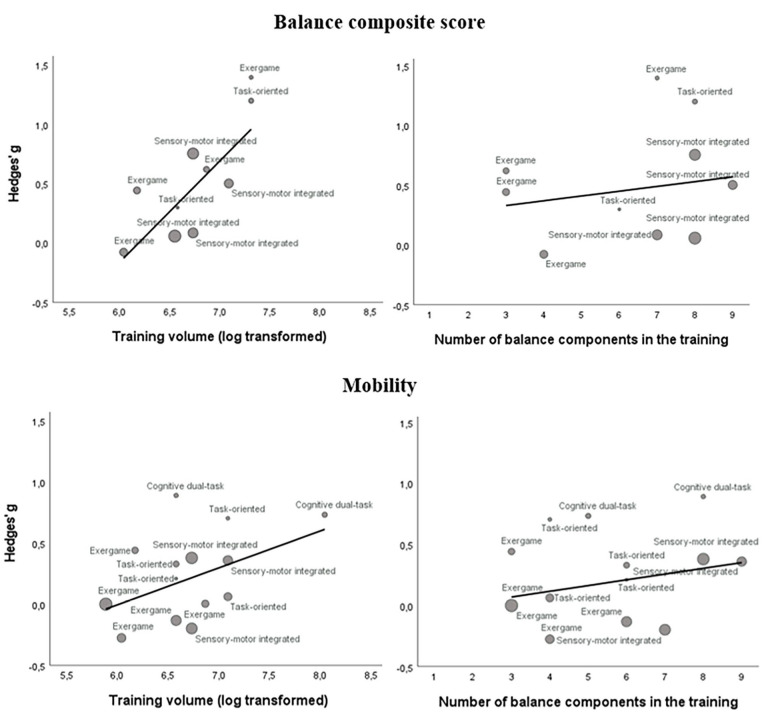
Bubble plots on *training volume (log transformed)* and *number of balance components in the training* of all included studies labeled with training types (cognitive dual-task, sensory-motor integrated, task-oriented, and exergame) and grouped within the respective outcome categories *Balance composite score* and *Mobility*. The bubble size indicates the study weight.

Visual inspection of weighted scatter plots (Figure 2) indicated an association between *training volume* and ES when measured by the outcome categories *Balance composite score* and *Mobility*. No association was found between *Number of components in the training* and ES. Analyses were conducted with studies pooled across training types. Corresponding meta-regressions showed significant associations between training volume and ES for the outcome categories *Balance composite score* (β = 1.05 [SE = 0.31]; *p* < 0.01) and *mobility* (β = .47 [SE = 0.21]; *p* = .03; Supplemental Material).

#### Publication Bias

The outcome categories *Balance composite score* and *Mobility* showed symmetric distribution in Funnel plots, indicating no publication bias (Supplemental Material).

## Discussion

This systematic review and meta-analysis evaluated the effect of diverse balance training interventions on balance and gait outcomes and the association between intervention complexity and exercise effect in PwMS. Balance training interventions displayed small to large ES when evaluated with balance composite scores and mobility tests. In contrast, effects of balance training on gait speed and stepping, that are considered less complex outcomes, showed no effects. Training volume was significantly associated with training effect, irrespective of training type. Contrary to our expectations, intervention complexity derived from the number of balance components included in the training did not emerge as a significant predictor. Notably, interventions employing a task-oriented training approach tended to yield the most favorable outcomes.

In the present review, balance training was constrained to interventions that in more than 50% of the session time directly target balance, with exclusion of interventions like resistance training,^53,54^ aquatic exercise,^55,56^ and aerobic training.^57^ Compared to previous reviews with broader criteria regarding types of interventions,^25,58^ this review included studies with a primary focus on balance. However, various kinds of training can be argued to impact neural pathways associated with postural balance^25^ and therefore likely capable of enhancing balance. This makes investigation of exposures that particularly impacts balance difficult.

Similarly to the association between intensity and physiologic gains in resistance and aerobic training, it is expected that intensity also matters in balance training. However, in the absence of an expression of intensity based on the relative level of challenge, the complexity of the applied interventions (based on the number of balance components included in the training) was proposed as a proxy for intensity in our previous review.^20^ It was hypothesized that interventions with a higher level of complexity would have a larger effect on balance compared to interventions with a lower level. This hypothesis was not confirmed in any outcome category and may be explained by an uncertainty of how challenging the respective balance interventions were relative to the participants capacity.

The categorization of training types was derived from the descriptions of interventions in the studies included. Of note, it is uncertain whether the authors of the included studies would concur with the categorization applied in present review and/or if the applied categories accurately capture the actual intervention content. Despite this uncertainty, the meta-analysis results suggest a more pronounced effect from task-oriented training compared to exergame and sensory-motor integration training, when assessed by composite scores (ES = 0.76 [95% CI = –0.13 to 1.64]) and mobility tests (ES = 0.29 [95% CI = –0.09 to 0.68]). This observation aligns with existing recommendations stating the importance of specificity in training.^59^

Furthermore, while an intervention may target a multitude of balance components, it may not be perceived as more challenging. If intensity in balance training is compared to intensity in aerobic or resistance training, achieving large effects often demand near-maximal loading, this would translate into a high level of challenge in balance training.

In contrast to the proposed intensity measure that considered intervention content, the quantity of training sessions was associated with ES measured by a balance composite score or a mobility test, irrespective of the training type. This result is in line with the review by Corrini et al,^25^ which reported a significant association between effect and session time and a near-significant association with total training volume. In support, a review by Gunn et al^58^ demonstrated an association between the effect of balance training and exercise volume (minutes/week). Associations in these reviews are, however, derived from studies with a broader definition of balance training.

Despite the attempt to detect characteristics, such as intensity, of balance training interventions that were associated with effect, this review did not succeed in doing so. Due to confusion related to the reporting of intensity in balance training, the authors of this review encourage further discussion on how intensity is best defined and implemented in future balance training studies.

Meta analysis on subgroup level showed a moderate to large ES for balance training delivered as either exergaming (ES = 0.55 [95% CI = –0.03 to 1.12]), sensory-motor integrated training (ES = 0.33 [95% CI = –0.02 to 0.69]), or task-oriented training (ES = 0.76 [95% CI = –0.13 to 1.64]), when evaluated with a balance composite score. These subgroup results were not significant but tended to be. The outcome category *balance composite score* was derived from Bergs Balance Scale in 7 studies (8 interventions),^30,31,33,34,43,44,51^ and from the Mini-BESTest^60^ in 2 studies.^47,50^ The Berg Balance Scale assesses mainly the ability to sustain static position under escalating level of difficulties but does not include gait components, balance reactions, or dual-tasking. Such absence of an ambulation component may explain the somewhat uniform effects observed across training types despite content diversity in the interventions. Based on this, measurements that include ambulation components are recommended.

When the subgroup effect of different training types was measured by a mobility outcome, where 13 out of 14 interventions utilized the Timed Up & Go test, exergame failed to demonstrate an effect, while training with a more functional approach exhibited ESs ranging from 0.19 in sensory-motor integrated training to 0.81 in cognitive dual-task training. The result in the present review likely reflects the importance of task-specificity when designing training, as stationary training does not show an effect on outcome measures that capture balance while moving around.^59^ However, it’s important to note that these results are derived from a limited number of studies within each subgroup, with only the effects from cognitive dual-task training^32,46^ reaching statistical significance.

When assessing the effect of balance training on gait speed, all training types yielded negligible and insignificant results. This might be explained by the hypothesis that balance training enhances adaptive and reactive strategies by controlling subtle yet precise movement. Improvement of balance may not significantly manifest in outcomes assessing habitual movement patterns prioritizing speed and force over adaptation and precision. Moreover, balance exercises may not impose sufficient loads on peripheral structures to impact the prerequisites for achieving high gait speed.

The most prominent finding from the risk-of-bias assessment was that only 2 studies (11%) reported activity monitoring in the control groups.^35,46^ This introduces the risk of making false conclusions on causal effect of the interventions as the activity type and level in the control groups are unknown. Uncertainty of how balance is impacted by different types of training affecting pathways associated with balance makes reporting of activity in the compared groups essential in future studies.

### Clinical and Research Implications

The meta-regression results from the present review align with a prior review^25^ recommending interventions with high training volume to ensure a sufficient stimulus for achieving training effect. Our results recommend the use of outcome measures encompassing multiple balance components evaluating adaptive, reactive, and ambulation properties, such as the Mini-BESTest^61^ to detect effect on balance performance in general and not just specifically for PwMS. Moreover, gait tests only assessing maximal walking speed (eg, 25-Foot Walk Test) may not capture potential improvements related to balance that would be captured by more complex gait outcomes (eg, Six Spot Step Test). Inconsistent and insufficient reporting of both intervention content and applied progression model, poses a challenge to comparability, when attempting to identify the key elements of balance training. Furthermore, a persistent absence of standardized methods applied to measure and report intensity and quality in balance training hinders the collective understanding and development of balance training interventions. Consequently, specific contributions of factors such as difficulty level, type of sensory stimuli, and cognitive processing requirements to the enhancement of functional balance remain unclear. Future balance training research in PwMS should therefore provide detailed descriptions of the applied interventions to advance the field.

The recognized knowledge gap regarding the effects of balance training and its association to dose, intensity, and training type is not restricted to balance training in PwMS, but is generalizable to various patient groups. However, since the effects of balance training partly might be explained by cognitive adaptation and learning, the cause of balance impairment in different patient groups may restrict the generalizability of effects.

### Strengths and Limitations

This review included 18 studies, representing 4 different balance training types, and involving more than 900 PwMS. Further, this is the first meta-analysis making the ambitious attempt to apply a definition based on a minimum of balance training content that specifically focuses on improving balance. In addition, this review defined intensity in balance training without confusing this with training volume parameters, that is, duration, frequency, and session time. However, a limitation is that certain training types only applied a few studies (ie, cognitive dual-task training was only applied in 2 studies^32,46^). The limited number of studies prohibited the assessment of additional covariates in the meta-regression. Consequently, it was not possible to determine whether the observed effect across all studies and within the respective training types depended on intervention duration and/or disability status.

The meta-analysis used ES that is a dimensionless measure of effect allowing pooling across outcome measures. However, the size of an effect relies on ratio between effect and sample distribution which complicates interpretation. The small ES found, might reveal a trend toward an effect, but it is hard to translate or make a useful interpretation of the results to outcome measures and functional tests used in clinical contexts.

Due to notable discrepancies between the calculated effects in our primary analysis and those reported by the individual studies, a supplementary meta-analysis was performed, using post-test values adjusted for baseline values (see Supplemental Material). Of note, in both methodological approaches, the analysis found the composite scores to be superior in detecting a change in balance control and that measures of mobility that evaluate more elements than speed alone are recommendable. These findings therefore add support to the validity of the main analyses.

## Conclusions

In this review, balance training was categorized into the training types: cognitive dual-task; exergame; sensory-motor integrated; and task-oriented balance training. The training types demonstrated comparable beneficial effects on balance when measured by multi-dimensional balance composite scores and mobility tests but not in terms of gait speed.

Intervention complexity determined from the number of balance components challenged in the training was not associated with an effect on balance. However, dynamic and cognitively challenging balance training tended to improve complex mobility, in contrast to exergame training. Interventions employing a task-oriented training approach tended to yield the most favorable outcomes.

Training volume across training types was associated with effect on balance. However, a definition of intensity in balance training is needed for evaluation of its impact on the effect of balance interventions.

## Supplemental Material

sj-docx-1-nnr-10.1177_15459683241273402 – Supplemental material for Effects of Balance Exercise Interventions on Balance-Related Performance in People With Multiple Sclerosis: A Systematic Review and a Meta-Analysis of Randomized Controlled Trials Supplemental material, sj-docx-1-nnr-10.1177_15459683241273402 for Effects of Balance Exercise Interventions on Balance-Related Performance in People With Multiple Sclerosis: A Systematic Review and a Meta-Analysis of Randomized Controlled Trials by Andreas Wallin, Sverker Johansson, John Brincks, Ulrik Dalgas, Erika Franzén and Jacob Callesen in Neurorehabilitation and Neural Repair
