# Supplementary material for: Effects of Balance Exercise Interventions on Balance-Related Performance in People With Multiple Sclerosis: A Systematic Review and a Meta-Analysis of Randomized Controlled Trials
Source: Neurorehabil Neural Repair. 2024 Aug 20;38(10):775–90. doi: 10.1177/15459683241273402 (PMC11490070; doi:10.1177/15459683241273402)
Supplement: sj-docx-1-nnr-10.1177_15459683241273402 – Supplemental material for Effects of Balance Exercise Interventions on Balance-Related Performance in People With Multiple Sclerosis: A Systematic Review and a Meta-Analysis of Randomized Controlled Trials [file sj-docx-1-nnr-10.1177_15459683241273402.docx]

| **Outcome category: Balance composite score** | | | | | | | | | | | |
| --- | --- | --- | --- | --- | --- | --- | --- | --- | --- | --- | --- |
| **Training type** | **Author** | **Outcome measure** | **Intervention** | |  | **Control** | | **Weight (%)** | **SMD (CI 95%)** | **Favors** | |
|  |  |  | **n** | **Change_post-pre_ (SD_post_)** |  | **n** | **Change_post-pre_ (SD_post_)** |  |  | **Control** | **Intervention** |
| **Exergame** | Yazgan et al. 2020 | BBS | 15 | 5.8 (4.3) |  | 15 | .9 (7.1) | 9.0. | .81 (.06; 1.55) | 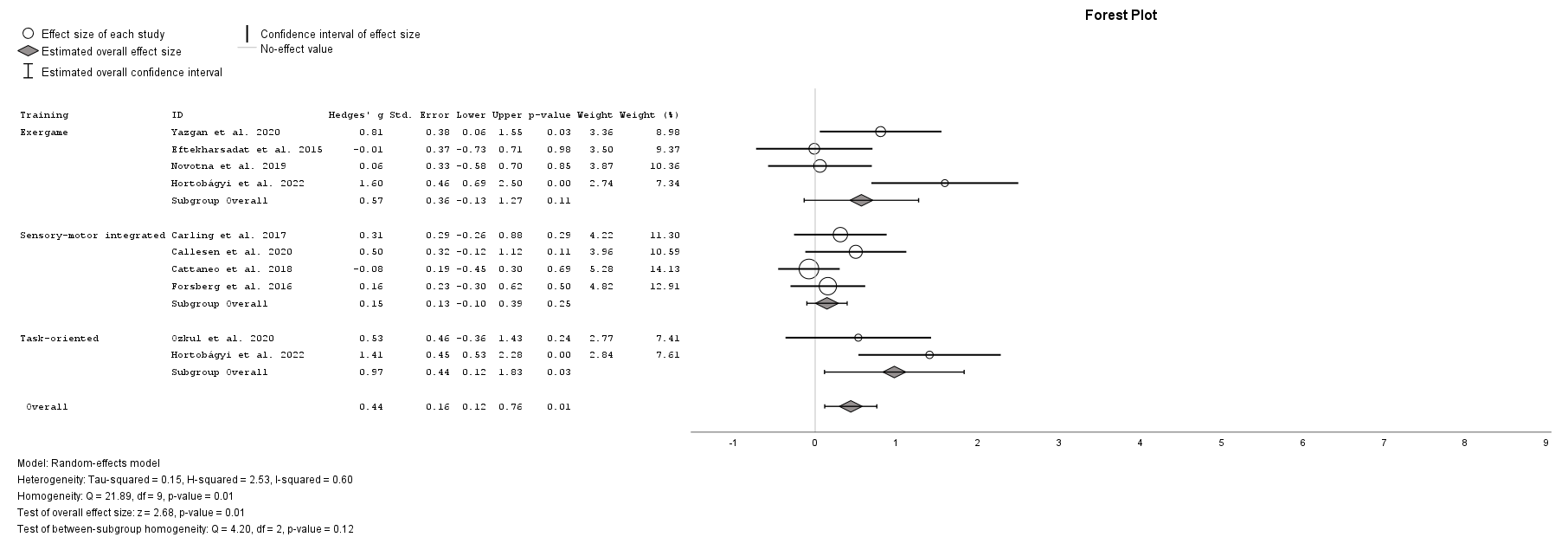 | |
|  | Eftekharsadat et al. 2015 | BBS | 15 | .2 (3.2) |  | 15 | .3 (9.0) | 9.4 | –.01 (–.73; .71) |  |  |
|  | Novotna et al. 2019 | MBT | 23 | 1.13 (6.0) |  | 16 | .8 (3.8) | 10.4 | –.06 (–.58; .70) |  |  |
|  | Hortobágyi et al. 2022 | BBS | 14 | 6.1 (3.8) |  | 12 | –.2 (3.9) | 7.3 | 1.60 (.69; 2.50) |  |  |
|  | **Subgroup overall** |  | **67** |  |  | **58** |  | **36.1** | .**57 (–.13; 1.27)** |  |  |
|  |  |  |  |  |  |  |  |  |  |  |  |
| **Sensory-motor integrated** | Carling et al. 2017 | BBS | 23 | 4.3 (11.5) |  | 25 | .6 (11.9) | 11.3 | .31 (–.26; .88) |  |  |
|  | Callesen et al. 2020 | MBT | 24 | 4.1 (6.4) |  | 18 | .9 (6.1) | 10.6 | .50 (–.12; 1.12) |  |  |
|  | Cattaneo et al. 2018 | BBS | 78 | 2.6 (5.3) |  | 41 | 3.0 (5.1) | 14.1 | –.08 (–.45; .30) |  |  |
|  | Forsberg et al. 2016 | BBS | 35 | 2.6 (4.5) |  | 38 | 1.6 (7.6) | 12.9 | .16 (–.30; .62) |  |  |
|  | **Subgroup overall** |  | **160** |  |  | **122** |  | **48.9** | **.15 (–.10; .39)** |  |  |
|  |  |  |  |  |  |  |  |  |  |  |  |
| **Task-oriented** | Ozkul et al. 2020 | BBS | 10 | 3.2 (5.8) |  | 10 | –.2 (6.4) | 7.4 | .53 (–.36; 1.43) |  |  |
|  | Hortobágyi et al. 2022 | BBS | 14 | 3.8 (1.1) |  | 12 | –.2 (3.9) | 7.6 | 1.41 (.53; 2.28) |  |  |
|  | **Subgroup overall** |  | **24** |  |  | **22** |  | **15.0** | **.97 (.12; 1.83)** |  |  |
|  |  |  |  |  |  |  |  |  |  |  |  |
|  | **Total overall** |  | **251** |  |  | **202** |  |  | **.44 (.18; .74)** |  |  |
|  |  |  |  |  |  |  |  |  | |  |  |
|  |  |  |  |  |  |  |  | Overall effect: *p* = .01 | | Heterogeneity: *I*^2^ = 60% | |

**Supplementary**

SD=Standard Deviation; SMD=Standardized mean difference; CI=Confidence Interval.

**Sup. Fig. 1.** Meta-analysis of the adjusted effect (post-measures minus pre-measures) of balance training on the outcome category *Balance composite score* in subgroups of training types.

| **Outcome category: Gait speed outcomes** | | | | | | | | | | | |
| --- | --- | --- | --- | --- | --- | --- | --- | --- | --- | --- | --- |
| **Training type** | **Author** | **Outcome measure** |  | **Intervention** |  | **Control** | | **Weight (%)** | **SMD (CI 95%)** | **Favors** | |
|  |  |  | **n** | **Change_post-pre_ (SD_post_)** |  | **n** | **Change_post-pre_ (SD_post_)** |  |  | **Control** | **Intervention** |
| **Exergame** | Nilsagaard et al. 2013 | 25FWT | 41 | .3 (3.0) |  | 39 | –.1 (3.8) | 19.9 | .11 (–.33; .55) | 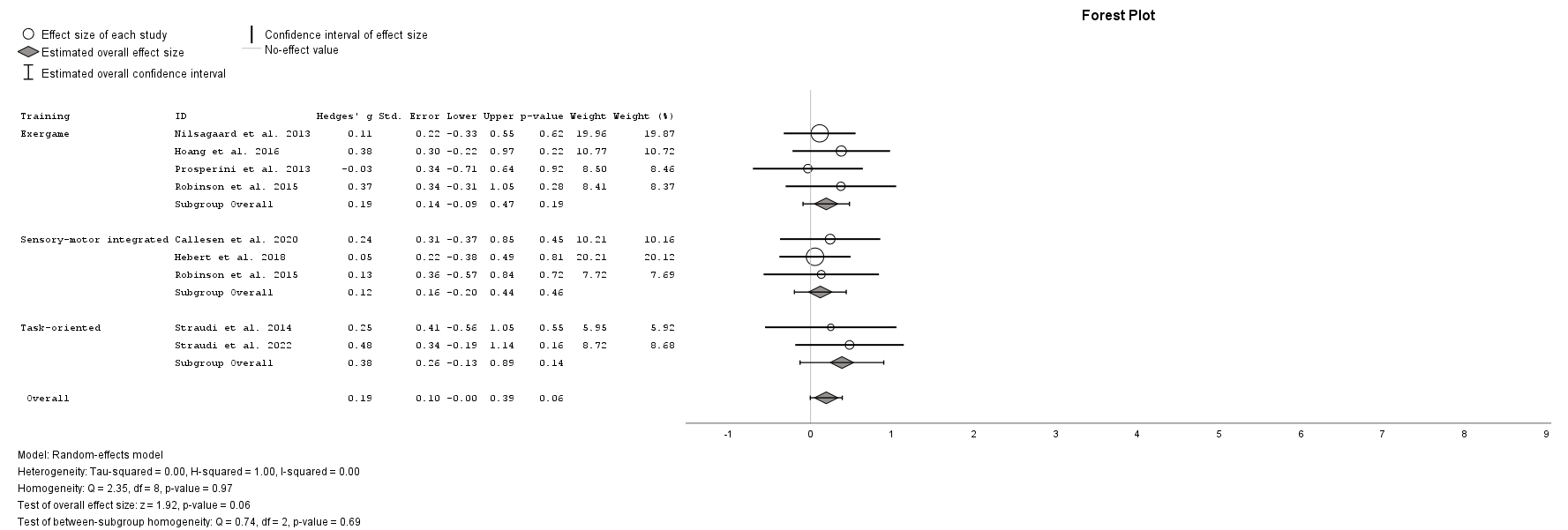 | |
|  | Hoang et al. 2016 | 10MWT | 23 | 2.0 (4.0) |  | 21 | .3 (4.9) | 10.7 | .38 (–.22; .97) |  |  |
|  | Prosperini et al. 2013 | 25FWT | 17 | .7 (2.8) |  | 17 | .8 (3.0) | 8.5 | –.03 (–.71; .64) |  |  |
|  | Robinson et al. 2015 | GAITRite^*^ | 20 | 11.9 (27.1) |  | 15 | .0 (36.5) | 8.4 | .37 (–.31; 1.05) |  |  |
|  | **Subgroup overall** |  | **101** |  |  | **92** |  | **47.5** | **.19 (–.09; .47)** |  |  |
|  |  |  |  |  |  |  |  |  |  |  |  |
| **Sensory-motor integrated** | Callesen et al. 2020 | 25FWT^*^ | 24 | .11 (.3) |  | 18 | .0 (.5) | 10.2 | .24 (–.37; .85) |  |  |
|  | Hebert et al. 2018 | 25FWT | 39 | .2 (2.0) |  | 42 | .1 (2.0) | 20.1 | .05 (–. 38; .49) |  |  |
|  | Robinson et al. 2015 | GAITRite^*^ | 16 | 4.1 (23.9) |  | 15 | .0 (36.5) | 7.7 | .13 (–.57; .84) |  |  |
|  | **Subgroup overall** |  | **79** |  |  | **75** |  | **38.0** | **.12 (–.20; .44)** |  |  |
|  |  |  |  |  |  |  |  |  |  |  |  |
| **Task-oriented** | Straudi et al. 2014 | 10MWT^*^ | 12 | .1 (.2) |  | 12 | –.0 (.2) | 5.9 | .25 (–.56; 1.05) |  |  |
|  | Straudi et al. 2022 | 10MWT^*^ | 18 | .1 (.2) |  | 18 | .0 (.2) | 8.7 | –.48 (–.19; 1.14) |  |  |
|  | **Subgroup overall** |  | **30** |  | | **30** |  | **14.7** | **.38 (**–**.13; .89)** |  |  |
|  |  |  |  |  | |  |  |  |  |  |  |
|  | **Total overall** |  | **210** |  | | **197** |  |  | **.19 (–.00; .39)** |  |  |
|  |  |  |  |  | |  | |  |  |  |  |
|  |  |  |  |  | |  | | Overall effect: *p* = .06 | | Heterogeneity: *I*^2^ = 0% | |

SD=Standard Deviation; SMD=Standardized mean difference; CI=Confidence Interval; 25FWT=25-Foot Walk Test measured in seconds; 10MWT=10-Meter Walk Test measured in seconds. ^*^=measured in meters/second.

**Sup. Fig. 2.** Meta-analysis of the adjusted effect (post-measures minus pre-measures) of balance training on the outcome category *Gait speed outcomes* in subgroups of training types.

| **Outcome category: Mobility** | | | | | | | | | | | |
| --- | --- | --- | --- | --- | --- | --- | --- | --- | --- | --- | --- |
| **Training type** | **Author** | **Outcome measure** | **Intervention** | |  | **Control** | | **Weight (%)** | **SMD (CI 95%)** | **Favors** | |
|  |  |  | **n** | **Change_post-pre_ (SD_post_)** |  | **n** | **Change_post-pre_ (SD_post_)** |  |  | **Control** | **Intervention** |
| **Cognitive dual-task** | Ozkul et al. 2023 | TUG | 13 | .8 (.6) |  | 13 | –.2 (.8) | 3.4 | 1.37 (.50; 2.24) | 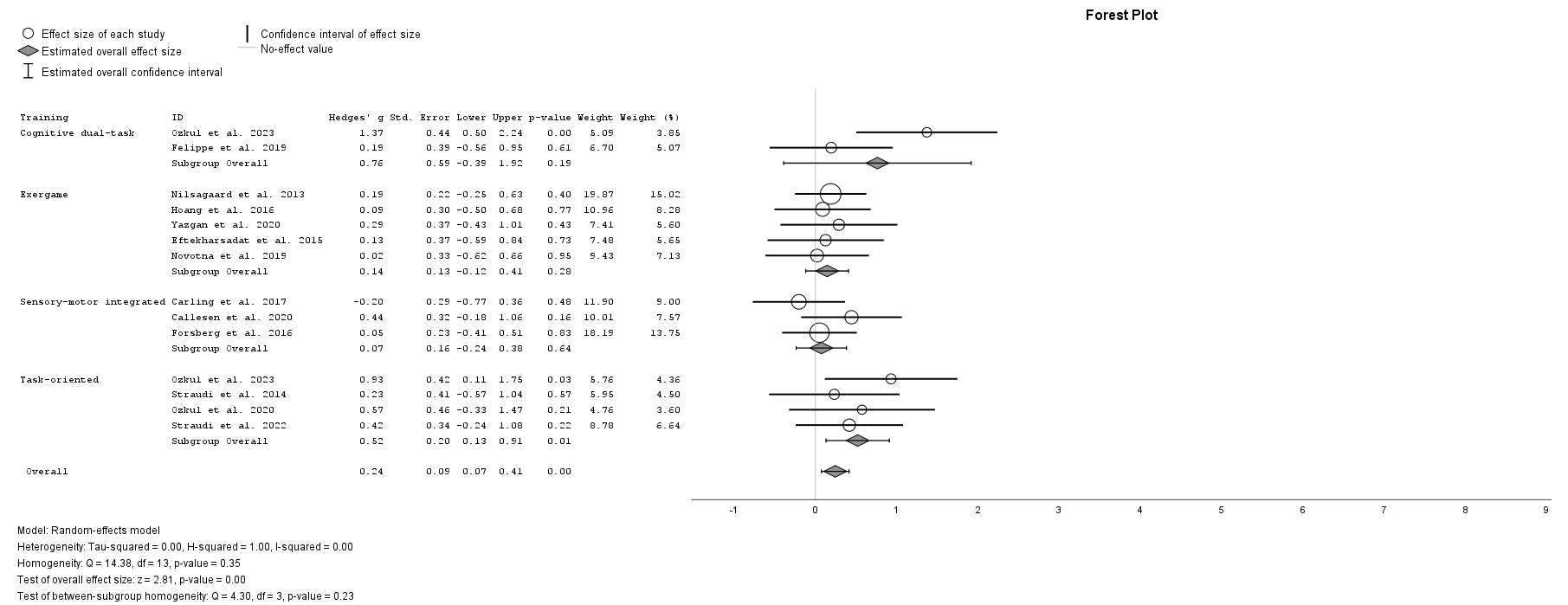 | |
|  | Felippe et al. 2019 | TUG | 13 | .6 (5.0) |  | 14 | –.8 (7.6) | 5.1 | .19 (–.56; .95) |  |  |
|  | **Subgroup overall** |  | **26** |  | | **27** |  | **8.5** | **.76 (.39; 1.92)** |  |  |
|  |  |  |  |  | |  | |  |  |  |  |
| **Exergame** | Nilsagaard et al. 2013 | TUG | 41 | 1.0 (5.9) |  | 39 | –.1 (5.7) | 15.0 | –.19 (–.25; .63) |  |  |
|  | Hoang et al. 2016 | TUG | 23 | .8 (4.3) |  | 21 | .4 (4.6) | 8.3 | –.09 (–.50; .68) |  |  |
|  | Yazgan et al. 2020 | TUG | 15 | 1.5 (4.9) |  | 15 | –.1 (5.9) | 5.6 | .29 (–43; 1.01) |  |  |
|  | Eftekharsadat et al. 2015 | TUG | 15 | .7 (2.2) |  | 15 | –.2 (9.3) | 5.7 | .13 (–.59; .84) |  |  |
|  | Novotna et al. 2019 | TUG | 23 | .6 (9.9) |  | 16 | .45 (5.1) | 7.1 | –.02 (–.62; .66) |  |  |
|  | **Subgroup overall** |  | **117** |  | | **106** |  | **41.7** | –**.14 (–.12; .41)** |  |  |
|  |  |  |  |  | |  | |  |  |  |  |
| **Sensory-motor integrated** | Carling et al. 2017 | TUG | 23 | .3 (23.6) |  | 25 | 4.8 (20.0) | 9.0 | –.20 (–.77; .36) |  |  |
|  | Callesen et al. 2020 | SSST | 24 | 1.1 (2.0) |  | 18 | .2 (2.0) | 7.6 | .44 (–.18; 1.06) |  |  |
|  | Forsberg et al. 2016 | TUG | 35 | –.5 (11.4) |  | 38 | –1.0 (8.3) | 13.8 | .05 (–.41; .51) |  |  |
|  | **Subgroup overall** |  | **82** |  | | **81** |  | **30.4** | **.07 (–.24; .38)** |  |  |
|  |  |  |  |  | |  | |  |  |  |  |
| **Task-oriented** | Ozkul et al. 2023 | TUG | 13 | .47 (.6) |  | 13 | –.18 (.8) | 4.4 | .93 (.11; 1.75) |  |  |
|  | Straudi et al. 2014 | TUG | 12 | .59 (1.9) |  | 12 | –.13 (3.8) | 4.5 | .23 (–.57; 1.04) |  |  |
|  | Ozkul et al. 2020 | TUG | 10 | 1.4 (2.7) |  | 10 | –.2 (2.8) | 3.6 | .57 (–.33; 1.47) |  |  |
|  | Straudi et al. 2022 | TUG | 18 | 1.4 (2.3) |  | 18 | .54 (1.4) | 6.6 | .42 (–.24; 1.08) |  |  |
|  | **Subgroup overall** |  | **53** |  | | **53** |  | **19.1** | **.52 (–.13; .91)** |  |  |
|  |  |  |  |  | |  | |  |  |  |  |
|  | **Total overall** |  | **278** |  | | **267** |  |  | **.24 (.07; .41)** |  |  |
|  |  |  |  | | |  | |  |  |  |  |
|  |  |  |  | | |  | | Overall effect: *p* = .00 | | Heterogeneity: *I*^2^ = 0% | |

SD=Standard Deviation; SMD=Standardized mean difference; CI=Confidence Interval; TUG=Timed Up&Go measured in seconds; SSST=Six Spot Step Test measured in 1/seconds.

**Sup. Fig. 3.** Meta-analysis of the adjusted effect (post-measures minus pre-measures) of balance training on the outcome *Mobility* in subgroups of training types.

| **Outcome category: Stepping** | | | | | | | | | | | |
| --- | --- | --- | --- | --- | --- | --- | --- | --- | --- | --- | --- |
| **Training type** | **Author** | **Outcome measure** | **Intervention** | |  | **Control** | | **Weight (%)** | **SMD (CI 95%)** | **Favors** | |
|  |  |  | **n** | **Change_post-pre_ (SD_post_)** |  | **n** | **Change_post-pre_ (SD_post_)** |  |  | **Control** | **Intervention** |
| **Exergame** | Nilsagaard et al. 2013 | FSST | 41 | –.4 (16.5) |  | 39 | 1.8 (10.7) | 67.4 | –.16 (–.59; .28) | 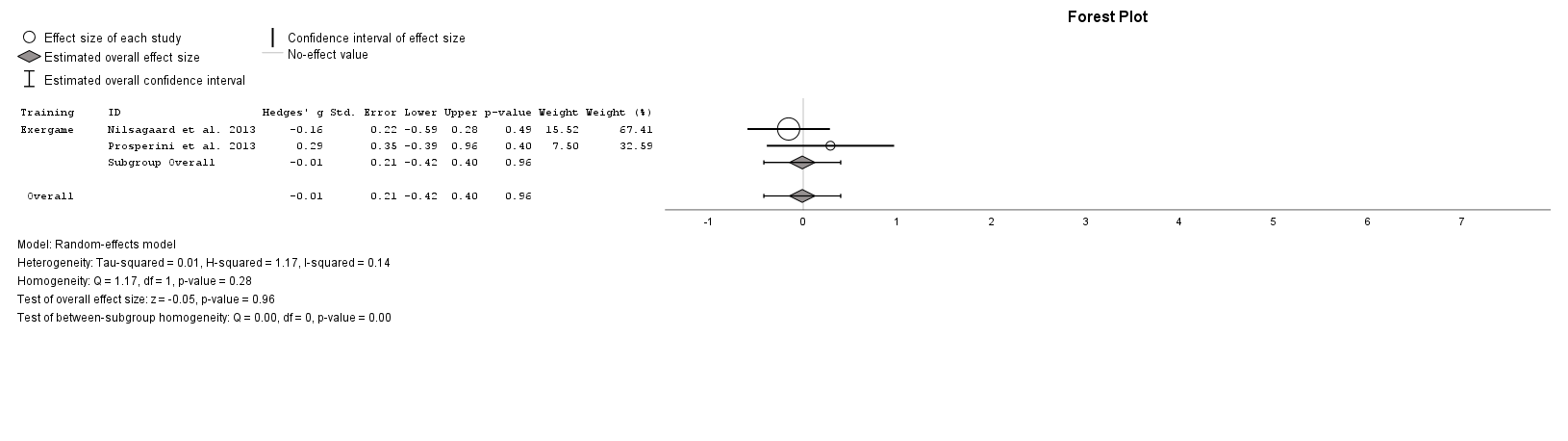 | |
|  | Prosperini et al. 2013 | FSST | 17 | 2.7 (10.1) |  | 17 | –.2 (9.5) | 32.6 | .29 (–.39; .96) |  |  |
|  | **Subgroup overall** |  | **58** |  | | **56** |  | **100.0** | **.01 (**–**.42; .40)** |  |  |
|  |  |  |  | | |  | |  |  |  |  |
|  | **Total overall** |  | **58** |  | | **56** |  |  | **–.01 (**–**.42; .40)** |  |  |
|  |  |  |  | | |  | |  |  |  |  |
|  |  |  |  | | |  | | Overall effect: *p* = .96 | | Heterogeneity: *I*^2^ = 14% | |

SD=Standard Deviation; SMD=Standardized mean difference; CI=Confidence Interval; FSST=Four Square Step Test measured in seconds.

**Sup. Fig. 4.** Meta-analysis of the adjusted effect (post-measures minus pre-measures) of balance training on the outcome *Stepping* in subgroups of training types.

**Sup. Table 1.** Univariate meta-regression including *training volume (logarithmic)* and *number of components in the training* of all included studies grouped within the respective outcome categories Balance composite score and Mobility.

| **Moderator** |  | **Outcome category** | | | | |
| --- | --- | --- | --- | --- | --- | --- |
|  |  | **Balance composite score** | |  | **Mobility** | |
|  |  | **β (SE)** | ***p*** |  | **β (SE)** | ***p*** |
| **Training volume (logarithmic)** |  | 1.05 (.31) | <.01 |  | .47 (.21) | .03 |
| **Number of balance components** |  | .03 (.86) | .69 |  | .05 (.07) | .77 |


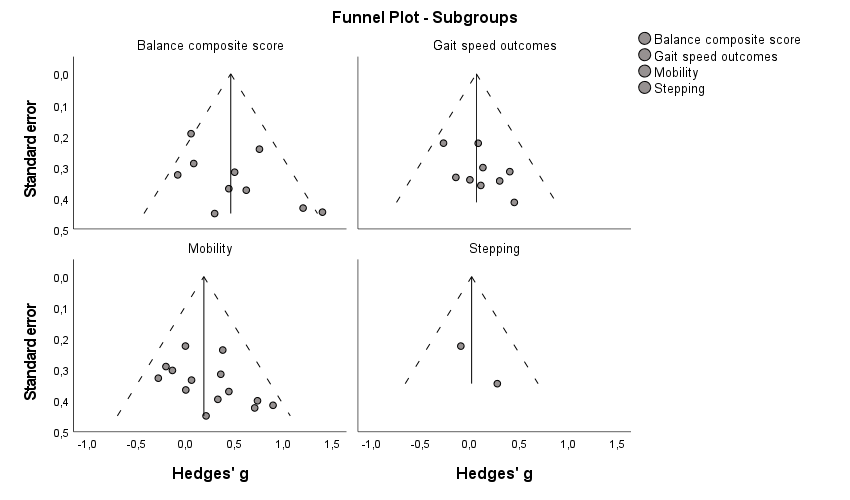


**Sup. Fig 5.** Funnel plots of all included studies grouped within the respective outcome categories *Balance composite score*, *Gait speed outcomes*, *Mobility*, and *Stepping*.
